# Supplementary material for: Diagnosis and Management of Catastrophic Antiphospholipid Syndrome and the Potential Impact of the 2023 ACR/EULAR Antiphospholipid Syndrome Classification Criteria
Source: Antibodies (Basel). 2024 Mar 12;13(1):21. doi: 10.3390/antib13010021 (PMC10967298; doi:10.3390/antib13010021)
Supplement: Supplementary file 1 [file antibodies-13-00021-s001.zip › antibodies-2829052-supplementary.pdf]

SUPPLEMENTARY MATERIAL

Table S1. Epidemiology of the Catastrophic antiphospholipid syndrome according to the literature

Diagnosis	Primary CAPS	60 %
	SLE-associated CAPS	30 %
	Others	10 %
Precipitating factors	Infections	30-50 %
	Surgery	17 %
	Malignancy	16 %
	Contraceptive	10 %
	Pregnancy-related	8-22 %
	Discontinuation of AC	unknown
	SLE flare	3 %
	Others	20 %
Mortality rate	Overall CAPS	37 %
	Primary CAPS	33 %
	SLE-associated CAPS	48 %
Laboratory features	Thrombocytopenia	67 %
	Schistocytes	22 %
	TMA [a]	14 %
	DIC [b]	11 %
	Lupus anticoagulant	83 %
	aCL IgG	81 %
	aCL IgM	49 %
	aβ2GPI IgG	78 %
	aβ2GPI IgM	40 %
	Antinuclear antibodies	57 %
	Anti-DNA antibodies	32 %

[a] TMA: Thrombotic microangiopathy defined as those patients with low platelet count, hemolysis features and schistocytes.

[b] DIC: Disseminated intravascular coagulation defined as those patients with low platelet count, d-dimer increase and low prothrombin time.

CAPS: catastrophic antiphospholipid syndrome; SLE: systemic lupus erythematosus; aCL: anticardiolipin antibodies; aβ2GP1: anti-beta-2 Gycoprotein 1 antibodies
